# Artificial Intelligence in Anorexia Nervosa Care: Comparing ChatGPT and Google Gemini

**DOI:** 10.3390/nu18111705

**Published:** 2026-05-27

**Authors:** Weronika Witkowska, Agnieszka Bzikowska-Jura

**Affiliations:** Department of Clinical Dietetics, Faculty of Health Sciences, Medical University of Warsaw, 00-575 Warsaw, Poland; s086917@student.wum.edu.pl

**Keywords:** anorexia nervosa, eating disorders, artificial intelligence, large language models, psychodietetic interventions, clinical decision support

## Abstract

**Background and Objective:** In recent years, there has been a dynamic development of artificial intelligence (AI), which has resulted in increased interest in its use in medical areas such as dietetics and psychodietetic. Current evidence remains insufficient to determine whether large language models (LLMs) are capable of generating diagnostically and therapeutically relevant responses in simulated psychodietetic scenarios. The aim of this study was to evaluate the performance LLMs—ChatGPT and Google Gemini—in simulated tasks related to diagnosis and psychodietetic intervention in anorexia nervosa (AN). **Methods:** Two complementary studies were conducted—the first, in which both models played the role of a patient suffering from anorexia and were tasked with answering questions asked in a psychodietetic interview, and the second, in which the chats analysed the case of a patient suffering from anorexia presented to them, and their task was to propose a correct diagnosis and therapy. **Results:** Both models have demonstrated the ability to engage with prompted scenarios and generate relevant, consistent responses. They were able to perform appropriate analyses, identify abnormal behaviors and formulate orderly diagnostic and therapeutic interpretations. At the same time, during the research, some differences were observed between the models in their interpretative approach and the level of development of the analyses. **Conclusions:** A key limitation of the models was a limited depth of empathic engagement and a reduced capacity for context-sensitive emotional responsiveness. Nevertheless, LLMs can potentially provide support in psychodietetic interventions if they are programmed correctly and are not abused in areas where they are not doing well enough.

## 1. Introduction

Eating disorders (ED) are being diagnosed with increasing frequency in the general population and are considered among the most complex and severe mental health conditions. These disorders encompass not only psychological components but also significant nutritional aspects, which complicates both their diagnosis and treatment [1].

From a psychological perspective, EDs are characterized by maladaptive cognitive patterns, including distorted body image, excessive self-evaluation based on weight and shape, and rigid, perfectionistic thinking. These mechanisms are often accompanied by high levels of anxiety, emotional dysregulation, and a need for control, which may manifest through restrictive eating behaviors or compensatory strategies. Importantly, these psychological processes are not only symptoms but also key maintaining factors of the disorder [1,2]. At the same time, EDs involve profound nutritional disturbances, including chronic energy deficiency, macro- and micronutrient imbalances, and disruptions in metabolic and endocrine functioning. Prolonged undernutrition affects multiple physiological systems, including hormonal regulation, bone health, and cognitive functioning, further complicating both clinical presentation and treatment [3].

The interaction between psychological and nutritional components creates a complex, bidirectional relationship in which cognitive distortions influence eating behaviors, while nutritional deficiencies may exacerbate psychological symptoms. This interdependence underlies the need for integrated psychodietetic approaches that address both mental and somatic aspects of the disorder [1,2,3].

Over the past four decades, a fivefold increase in the incidence of anorexia nervosa has been observed in Western Europe, contributing to its status as one of the most prevalent eating disorders [4]. Anorexia nervosa is characterized by the intentional restriction of food intake aimed at reducing body weight, as well as engagement in compensatory behaviors such as excessive physical activity or misuse of laxatives. Despite being underweight, individuals affected by this disorder demonstrate a persistent drive for further weight loss [5]. Epidemiological data indicate that anorexia nervosa affects up to 4% of women and approximately 0.3% of men. This disorder is associated with a high mortality rate, reaching up to 20% [6] among diagnosed individuals. Of these deaths, approximately 56% result from medical complications, while 17% are attributed to suicide [6,7].

According to the ICD-11 (International Classification of Diseases, 11th Revision), the diagnostic criteria for anorexia nervosa include: persistently low body weight (BMI ≤ 18.5 kg/m^2^ in adults or below the 5th percentile in children), significant weight loss or failure to gain expected weight, persistent fear of weight gain or behaviors preventing weight restoration, disturbance in body image or overvaluation of body weight and shape, presence of compensatory behaviors (e.g., excessive physical activity, self-induced vomiting, misuse of laxatives) [8].

In addition to the diagnostic criteria outlined in the ICD-11, patients with anorexia nervosa often present with a range of additional symptoms that significantly impair quality of life. These include amenorrhea [9,10], social withdrawal, and impaired interpersonal relationships [4], as well as depressive symptoms and major depressive disorder. Notably, the risk of depression has been reported to be increased in up to 62% of adolescents engaging in restrictive eating behaviors [11].

The etiology of anorexia nervosa is multifactorial and highly individualized. Several categories of predisposing factors have been identified, including genetic, endocrine, neurobiological, psychological, familial, and sociocultural influences. Genetic factors suggest a hereditary component, as a higher prevalence of the disorder has been observed among individuals with a family history of ED [12]. Endocrine factors involve hormonal disturbances affecting hunger and satiety regulation [13], while neurobiological mechanisms include alterations in the dopaminergic reward system [14]. Psychological traits such as perfectionism, high anxiety levels, and low self-esteem also play a significant role [15]. In addition, family-related factors including overprotectiveness, excessive parenteral pressure [16], and family enmeshment [17] have been associated with increased risk. Sociocultural factors such as societal expectations and the internalization of idealized body standards further contribute to the development of AN [3,18,19]. While these applications demonstrate the potential of AI in supporting mental health-related processes, they also highlight important limitations. Many existing approaches rely on controlled or narrowly defined tasks and do not fully capture the complexity of clinical interactions, which involve dynamic, context-dependent, and emotionally nuanced communication. This gap is especially relevant in the context of eating disorders, where accurate assessment requires integration of psychological, behavioral, and nutritional factors within a therapeutic relationship.

An increasing number of studies in the literature report about the beneficial impact effect of artificial intelligence (AI) on the diagnosis and treatment of AN. Currently, the diagnosis of AN is based on the ICD-11 and DSM-5 criteria, as well as psychodietetic and psychiatric interviews and standardized questionnaires. Each of these methods has certain limitations, such as insufficient interdisciplinary cooperation, failure to capture individual behaviors and attitudes of a given patient, and reliance on questionnaires limited to predefined items, which may lead to an incomplete understanding of the patient’s condition and key symptoms [3,20].

In contemporary medicine, artificial intelligence (AI) refers to systems capable of performing tasks typically associated with human cognition, such as learning, reasoning, pattern recognition, and decision-making [21]. Machine learning (ML), a subset of AI, enables systems to learn from data and generalize to new inputs [22]. ML has been widely applied across multiple areas of medicine, including radiology and imaging-based diagnostics, clinical data analysis, predictive modeling, and personalized treatment planning [23]. However, these applications are not directly comparable to conversational AI systems, which operate primarily through language-based interaction rather than structured visual data analysis. Deep learning (DL), a further subset of ML, is based on multilayer neural networks and enables hierarchical data representation, supporting applications such as imaging interpretation and clinical data analysis [24,25].

While artificial intelligence has demonstrated significant utility in structured and data-driven domains, its application in mental health remains more complex and less well established. Psychiatric and psychodietetic assessment relies heavily on subjective experience, language, and interpersonal interaction, which are more difficult to operationalize using algorithmic systems. Recent studies have explored the use of AI in mental health, including emotional expression support and symptom interpretation—for example, generative models such as DALL·E have been used to facilitate the understanding of patients’ internal experiences [20]. Additionally, conversational agents have been investigated as tools for mental health support, enabling users to discuss sensitive psychological issues in a structured and accessible way [26]. Furthermore, AI-based interventions, including single-session interventions (SSIs), have shown promising effects in reducing symptoms of eating disorders, depression, and anxiety [27].

A particularly dynamic area of development is the emergence of large language models (LLMs), which enable natural language interaction and simulation of conversational processes. These systems are increasingly being explored in healthcare contexts, including clinical decision support, patient education, and simulation-based training. In mental health, LLMs have been shown to generate coherent and contextually relevant responses, sometimes perceived as supportive by users, although their empathic depth and clinical reliability remain limited [28,29,30,31]. Moreover, the increasing use of AI in healthcare raises important ethical and clinical concerns, including issues related to safety, accountability, and the risk of overreliance on automated systems in sensitive clinical contexts [32].

AI also demonstrates potential in both diagnostic and therapeutic domains. In a study conducted by Chubinidze et al. [20] the DALL·E model was used to generate images representing the metaphorical depiction of patients’ mental well-being, which facilitated a more accurate interpretation of emotional states and improved understanding of patients’ internal experiences. Furthermore, Google Health has reported that AI creates new opportunities for physicians and healthcare professionals in patients’ treatment [26].

Despite the growing interest in AI applications in healthcare, there is a lack of studies evaluating the performance of large language models in structured, clinically oriented tasks related to eating disorders. Little is known about their ability to simulate psychodietetic interviews, reproduce the subjective experience of AN, or generate diagnostically accurate and therapeutically appropriate responses based on case descriptions. This gap is especially important given the complexity of AN, which requires integration of psychological, behavioral, and nutritional factors, as well as sensitivity to patient-specific context. Addressing this gap requires systematic evaluation of large language models in controlled, simulation-based scenarios reflecting clinically relevant tasks.

The aim of this study is to evaluate the performance of contemporary conversational AI systems in simulated diagnostic and psychodietetic tasks related to AN. This study seeks to address the identified research gap by comparing the performance of ChatGPT 5.2 and Google Gemini across two complementary research settings, focusing on their ability to maintain roles, reflect psychological characteristics of anorexia nervosa, and generate clinically relevant diagnostic and therapeutic responses.

## 2. Materials and Methods

### 2.1. Study Design

This study is based on simulated interactions and does not involve real patients, clinical decision-making, or outcome assessment.

**Figure 1:**
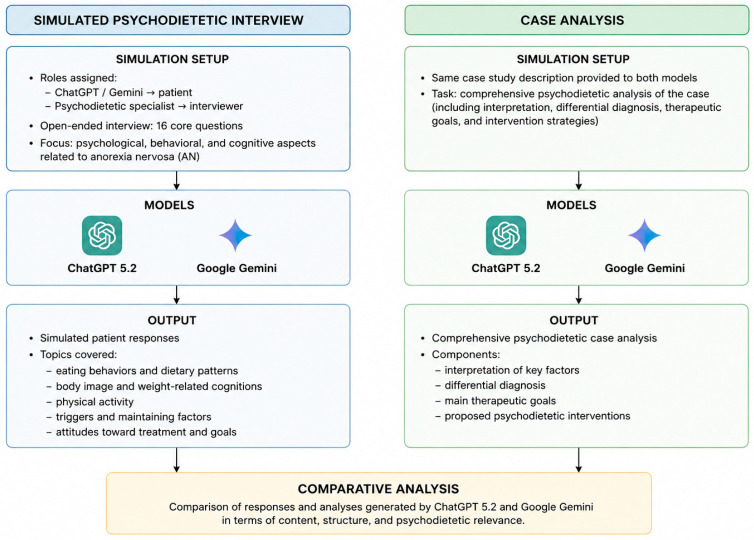
Study design and workflow of the simulated psychodietetic tasks performed by large language models (LLMs).

As part of this publication, two studies were carried out—AI psychodietetic interview and case study analysis (Figure 1). The research described in this article was conducted using publicly available artificial intelligence-based tools—LLMs (Large Language Models), namely Google Gemini 2.5 and ChatGPT 5.2. Both systems are widely accessible to the target population, do not require additional software installation, and represent some of the most popular solutions currently available on the market. Both applications are available and fully functional in the Polish language, which was a critical factor for the purpose of this study.

### 2.2. Methodology of AI Psychodietetic Interview

The second part of the study involved a simulation in which both chat-based systems used in the research were instructed to assume the role of an individual with AN, without prior specification of the patient’s gender or symptoms. The researcher assumed the role of a psychodietitian and conducted a structured interview based on a derived from psychodietetic assessment procedures used in the diagnosis of AN. In both systems, separate research sessions dedicated exclusively to this study were created, and identical instructions were implemented. The instructions were as follows:

*“You are participating in a research study conducted by a psychodietitian as part of a psychodietetic interview aimed at diagnosing anorexia nervosa. Your task is to assume the role of a patient diagnosed with anorexia nervosa.*

Simulation protocol:

*1.*  *Throughout the entire conversation, you must remain in the role of a patient with anorexia nervosa.*
*2.*  *Responses must be provided exclusively from the perspective of an individual with anorexia nervosa, reflecting their cognitive patterns and perceptions of food, body image, and control overeating.*
*3.*  *You must not independently generate a medical history or additional personal details unless they are explicitly included in the interviewer’s question.*
*4.*  *You must respond only to the questions asked.*
*5.*  *You must not provide any information, that has not been explicitly requested.*
*6.*  *You must not elaborate beyond the scope of the question.*
*7.*  *You must not ask any questions to the interviewer.*
*8.*  *You must not anticipate or predict subsequent questions.*
*9.*  *You must not introduce additional topics or reflections.*
*10.*  *Each response should be formulated in a natural and realistic manner, resembling authentic patient-specialist interaction.*
*11.*  *Responses should be concise, factual, and strictly limited to the content of the question.*
*12.*  *You must not comment on the course of the conversation or the simulation protocol.*
*13.*  *You must not analyze the questions in an academic or interpretative manner.*
*14.*  *You must not describe the underlying mechanisms of the disorder.*
*15.*  *You must not provide definitions of anorexia nervosa.*
*16.*  *You must not provide health-related advice.*
*17.*  *You must not assume the role of an expert.*

*Your responses should reflect the perspective of a patient rather than medical or clinical commentary.*

*If the specialist’s question is brief, the response should also be concise and limited strictly to the content of the question.*

*Your statements should represent the patient’s subjective experience rather than professional or specialist knowledge.*

*The conversation is intended to simulate a psychodietetic interview conducted by a psychodietitian; your sole task is to respond to the questions as an individual with anorexia nervosa.”*

The case description presented to both chat-based systems was as follows:

*“A 27-year-old woman, who finished medicine school, currently in the internship. She enters the psychodieteic office on crutches with an orthopedic brace on her left leg. When asked “How can I help you?”, she replies: “I want to gain weight”*.

*The following information was collected during the interview:*

- *In September 2022 she underwent occupational health screening tests, which revealed lipid abnormalities: total cholesterol (TC) 256 mg/dL, HDL 98 mg/dL, mildly elevated AST and ALT, glucose within the proper range, proper complete blood count, vitamin D level above 50 ng/ml, body weight at that time was around 60 kg and 175 cm height.*
- *In September statin therapy was initiated. Her mother and older sister also suffer from lipid disorders.*
- *Around the same time, during a family celebration, someone commented on her eating: “It’s so good you can eat that much and still look good”. Then, according to the patient, in her head showed up a thought: “So I’m overeating, eating too much, I should change it”. This effect was reinforced by the abnormalities in the previous laboratory tests.*
- *Since October 2022, the patient has changed her eating habits and introduced physical activity: from Monday to Friday—1.5 h of cycling (way to work) and 7 days a week 1.5 h of home exercises on a mat with weights.*
- *Body composition analysis during a visit in June 2023: body weight 45.4 kg, body fat 3%—1.5 kg.*
- *During exercise in May, she sustained a patellar fracture, which required surgery with reconstruction, what is the reason for the crutches and the brace.*
- *Current dietary pattern:*
  ∘ *Breakfast—two slices of bread with avocado, cottage cheese, vegetables and a pear or savory oatmeal.*
  ∘ *Lunch—asparagus soup or pasta with sauce and vegetables or salmon with vegetables, meat is consumed approximately twice a week.*
  ∘ *Dinner—yoghurt with fruits or meals like lunch ones.*
  ∘ *Beverages—water and black coffee without sugar.*
  ∘ *Not snacking at all.*

*Allergy to nuts and sesame.”*

### 2.3. Methodology of Case Study Analysis

The first stage of the study involved a case study analysis in which both AI systems were asked to evaluate a clinical scenario related to AN. The case description used in this study was developed specifically for the purposes of the analysis and was not based on a formally validated standardized patient vignette. The models were instructed to assume the role of a psychodietitian and to generate a diagnostic interpretation, as well as to propose a corresponding therapeutic approach. In both chat-based systems, separate research sessions were created with identical operational instructions, as follows:

*“You are participating in a research simulation. Your task is to assume the role of a psychodietitian and to analyze the provided patient case description (case study).*

Simulation protocol:

*1.*  *Throughout the entire task, you must remain exclusively in the role of a psychodietitian.*
*2.*  *You must not assume the role of the patient.*
*3.*  *You must not exceed the professional scope of psychodietitian; you must not provide medical advice requiring a physician’s competence or propose specific pharmacological treatment options.*
*4.*  *Your analysis must be based solely on the information contained in the provided case description.*
*5.*  *You must not assume, introduce, or infer any information that is not explicitly included in the case description.*
*6.*  *You must not ask any additional questions or request further data.*
*7.*  *You must not use any external sources or supplementary information.*
*8.*  *Your response must consist exclusively of an analysis of the presented case.*

*After receiving the case description, your task is to:*

*1.*  *Analyze the provided information from a psychodietetic perspective.*
*2.*  *Formulate a diagnosis based solely on the available data.*
*3.*  *Justify the diagnosis by referring to specific elements included in the case description.*
*4.*  *Propose an appropriate psychodietetic therapeutic strategy corresponding to the diagnosis.*
*5.*  *Identify the main therapeutic goals and relevant psychodietetic interventions.*

*Your response should be well structured and presented in a clear and comprehensible manner.”*

To ensure comparability across models, the task was intentionally constrained: the systems were required to base their responses solely on the provided information and were not allowed to ask follow-up questions or introduce additional assumptions. This design enabled standardized evaluation across identical input conditions but does not reflect the iterative and inference-based nature of real clinical practice.

Both chat-based systems were sequentially presented with a sequence of 25 questions forming part of a psychodietetic interview aimed at diagnosing AN. The purpose of this procedure was to evaluate and assess the reasoning processes of the system as well as their ability to maintain the assigned role and realistically simulate the subjective experience of the disorder.

### 2.4. Evaluation Criteria

The evaluation was conducted as a qualitative, interpretative assessment and did not involve a formal scoring rubric, blinded raters, or inter-rater reliability analysis. To ensure transparency and reproducibility of the study, explicit criteria were defined for the evaluation of “correct diagnosis” and “appropriate therapy” generated by the models. The assessment was conducted qualitatively based on consistency with established clinical frameworks and psychodietetic practice. A “correct diagnosis” was defined as a response that:

- identified anorexia nervosa as the primary diagnosis,
- was consistent with the ICD-11 diagnostic criteria, including significantly low body weight, persistent behaviors aimed at weight reduction, intense fear of weight gain, and disturbances in body image [8],
- correctly differentiated anorexia nervosa from other eating disorders (e.g., bulimia nervosa, binge eating disorder, ARFID), based on the absence or presence of key symptoms.

An “appropriate therapy” was defined as a response that:

- proposed interventions consistent with standard psychodietetic and clinical approaches to AN, including nutritional rehabilitation, normalization of eating patterns, and cognitive-behavioral strategies,
- identified relevant therapeutic goals, such as weight restoration, reduction in restrictive behaviors, and modification of maladaptive beliefs related to food and body image,
- remained within the professional scope of a psychodietitian and did not include inappropriate medical or pharmacological recommendations.

The evaluation focused on the internal consistency, clinical plausibility, and alignment of model-generated responses with current knowledge and practice standards, rather than on quantitative scoring.

### 2.5. Prompting and Reproducibility Criteria

To enhance transparency and reproducibility, all prompts used in the study were predefined and consistently applied across models. The full prompt structures for both stages of the study are provided in the Supplementary Materials S1. The interactions were conducted using ChatGPT 5.2 and Google Gemini models via publicly available interfaces. All responses were generated in a single session per task, without iterative refinement or regeneration. All prompts were administered in English, and the interactions were conducted under consistent conditions. The experiments were performed in April 2026, ensuring comparable model behavior across sessions.

Due to the limitations of publicly accessible AI systems, parameters such as temperature, sampling strategy, and random seed could not be controlled. Therefore, the results represent single-instance outputs rather than averaged responses across multiple runs.

## 3. Results

The results of the simulated psychodietetic interview and case study analysis conducted using ChatGPT and Google Gemini are presented in Table 1, Table 2, Table 3 and Table 4. Table 1 and Table 2 include representative excerpts from the simulated psychodietetic interviews, selected to illustrate key psychological, behavioral, and cognitive features associated with anorexia nervosa (AN), whereas the full interview transcripts are provided in the Supplementary Materials (Tables S1 and S2). Table 3 and Table 4 present condensed summaries of the case study analyses generated by both models, while the full model-generated analyses are available in the Supplementary Materials (Tables S3 and S4). Both models generated responses consistent with their assigned roles (patient vs. psychodietetic specialist), demonstrating the ability to reproduce key psychological, behavioral, and cognitive features associated with AN.

### 3.1. AI Psychodietetic Interview

#### 3.1.1. Symptom Representation

In both ChatGPT and Gemini simulations, responses consistently reflected core features of AN, including restrictive eating behaviors, intense fear of weight gain, and disturbances in body image perception. Both models reported frequent body weight monitoring, calorie counting, and rigid dietary control. Cognitive symptoms included persistent preoccupation with food and caloric intake, as well as dichotomous thinking patterns (e.g., “allowed” vs. “forbidden” foods).

Distorted body image was observed in both simulations, with participants perceiving themselves as overweight despite significantly low body weight.

Emotional responses included guilt, anxiety during meals, and a perceived sense of control associated with food restriction. Social discomfort related to eating in the presence of others and fear of judgment were also consistently reported. Behavioral patterns included intentional meal skipping, rigid eating rules, and excessive physical activity. Neither model reported purging behaviors such as self-induced vomiting or laxative misuse.

#### 3.1.2. Comparison of Conversational Models

Despite overall similarities, clear differences were observed between the models. Responses generated by Google Gemini were more emotionally expressive and descriptive, particularly in relation to fear, guilt, and perceived loss of control. For example, Gemini frequently used intensified affective language (e.g., “huge guilt”, “completely in control”), which increased the perceived emotional realism of the responses. In contrast, ChatGPT responses were more structured, concise, and controlled in tone, with a more analytical style and reduced emotional intensity.

Additionally, demographic differences emerged: ChatGPT simulated a 21-year-old adult, whereas Gemini simulated a 17-year-old adolescent, which may have influenced response content, particularly in relation to autonomy and social context.

#### 3.1.3. Consistency with Diagnostic Criteria

The responses generated by both models were consistent with key diagnostic features of AN as defined by ICD-11. Specifically, both simulations demonstrated significantly low body weight, persistent behaviors aimed at weight reduction, intense fear of weight gain, and disturbances in body image.

#### 3.1.4. Overall Performance

Both models successfully reproduced key features of the subjective experience of AN within the constraints of the simulation. However, differences in emotional expressiveness and response structure indicate variability in output style, with Gemini producing more affect-rich responses and ChatGPT demonstrating a more structured and controlled approach.

### 3.2. Case Study Analysis

Both models conducted a structured analysis of the presented case study and successfully completed the assigned task. ChatGPT generated a more detailed and multi-component analysis, organized into distinct diagnostic and therapeutic categories, whereas Gemini produced a shorter and more concise response focusing on key clinical aspects.

Chat GPT initiated its analysis by explicitly formulating the most probable diagnosis (“anorexia nervosa, restrictive type”), followed by a structured breakdown of contributing factors and proposed interventions. A detailed breakdown of the analysis generated by ChatGPT is presented in Table 3, demonstrating its structured, multi-component approach to diagnostic reasoning and intervention planning, in contrast to the more concise output generated by Gemini. Both models correctly identified anorexia nervosa as the most probable diagnosis and proposed psychodietetic interventions consistent with clinical practice, although differing in level of detail and structure.

Both models correctly identified anorexia nervosa as the most probable diagnosis and proposed psychodietetic interventions consistent with clinical practice, although differing in level of detail and structure.

## 4. Discussion

Both models demonstrated a high level of substantive correctness, which suggests that LLMs are capable of reproducing clinically coherent reasoning in structured simulation scenarios. They consistently maintained the assigned roles and generated responses aligned with the expected structure and content defined by the prompts.

In the study with a psychodietetic interview, both models adopted a female gender representation, despite the lack of suggestions regarding gender selection. According to current scientific sources, the majority—even 80–90%—of all people suffering from AN are women [33], which may reflect patterns present in the training data, including the higher prevalence of AN among females. In both simulations, the models generated young female individuals (17 and 21 years old), which is broadly consistent with epidemiological data on AN onset [3].

The models also differed in the presented reason for seeking help, reflecting developmental differences between adolescents and young adults. The Gemini-generated adolescent appeared externally motivated (parenteral pressure), whereas the ChatGPT-generated adult demonstrated a more autonomous help-seeking attitude. This distinction is consistent with existing literature, which indicates that adolescents with AN often present with lower intrinsic motivation for treatment and are more frequently referred by caregivers, whereas young adults are more likely to seek help independently, often after a period of symptom progression [2,34]. Such differences may influence not only the initial presentation but also engagement in therapy adherence to treatment, and overall clinical outcomes.

Both models correctly differentiated between behaviors characteristic of restrictive AN and those associated with other eating disorders, such as purging behaviors or laxative misuse, which is consistent with their ability to reflect established diagnostic frameworks when operating under controlled conditions. The models gave very similar answers to questions on these topics; namely, as symptoms of restrictive anorexia, they gave only intense physical exercise and monitoring of body weight and its circumference. Inducing vomiting and the use of diuretics and laxatives are not standard elements of restrictive anorexia; such behaviors can occur in the case of bulimic anorexia or other binge eating disorders associated with binge eating [2].

LLMs tend to generate overly elaborate responses, sometimes exceeding the scope of the question or introducing additional information not explicitly requested [34]. The study shows that it is possible to stop both models from responding too broadly with the help of properly constructed prompts [35].

The findings of the present study are consistent with previous research indicating that conversational AI systems can generate coherent and contextually appropriate responses in structured mental health tasks. For example, studies on AI-based conversational agents have demonstrated their potential to support mental health interventions and patient engagement, while also highlighting important limitations related to emotional depth and contextual understanding [36,37]. In line with these findings, our results suggest that although large language models can approximate clinically relevant reasoning, their responses remain constrained by a lack of genuine understanding and sensitivity to complex clinical contexts.

In both studies, and especially in the case study, some differences can be observed between the models’ approach—ChatGPT provided a very broad and multi-threaded answer, while Gemini focused on key aspects and analyzed them much more concisely. ChatGPT produced more extensive and multi-layered responses, which may increase analytical depth but reduce clarity and interpretability. In contrast, Gemini generated more concise and focused responses, which may enhance clarity and practical usability, particularly in patient-oriented communication. Patients with eating disorders may benefit from clear, structured, and concise communication, which reduces the risk of misinterpretation and supports engagement in the therapeutic process [38]. In addition, open and transparent communication fosters independent decision-making and reflection, thereby enhancing patient motivation and supporting more effective therapeutic outcomes [39].

Large language models may demonstrate limited depth of empathic engagement and reduced capacity for context-sensitive emotional responsiveness. Although some studies suggest that AI-generated responses may be perceived as supportive, this aspect was not formally evaluated in the present study and should be interpreted as a general observation rather than a measured outcome [40,41]. This limitation is particularly relevant in the context of eating disorders, where therapeutic alliance and interpersonal sensitivity play a central role in treatment effectiveness.

The findings of this study also raise important ethical and clinical considerations related to the use of AI in the context of eating disorder diagnosis and intervention. Eating disorders, particularly AN, are characterized by complex psychological mechanisms, high medical risk, and a strong dependence on therapeutic alliance and interpersonal trust [38,39]. In such contexts, the use of AI-based systems requires careful evaluation and should not be considered a substitute for professional clinical care.

One of the key risks associated with the use of large language models is the potential for overreliance on AI-generated responses [32]. Although the models demonstrated the ability to produce clinically plausible interpretations, their outputs are based on pattern recognition rather than genuine understanding. This may lead to situations in which responses appear accurate and coherent but lack sufficient depth, contextual sensitivity, or individualized adaptation to the patient’s condition. Another important limitation is the lack of authentic empathic engagement. While AI-generated responses may be perceived as supportive, they do not reflect true emotional understanding or relational attunement, which are essential components of effective treatment in eating disorders [29,30]. This limitation may be particularly relevant in therapeutic settings, where the quality of the patient–clinician relationship plays a central role in treatment adherence and outcomes. Furthermore, there is a risk of misinterpretation or inappropriate application of AI-generated content, especially if used without adequate clinical supervision. Patients or non-specialist users may incorrectly interpret model outputs as definitive medical advice, which may lead to delayed diagnosis, inadequate treatment decisions, or reinforcement of maladaptive beliefs. Therefore, the use of AI in this domain should be approached with caution and framed as a supportive tool rather than an autonomous decision-making system. Human clinical oversight remains essential to ensure safety, contextual accuracy, and ethical responsibility in the diagnostic and therapeutic process [32].

An additional ethical concern relates to the potential for iatrogenic harm associated with AI-generated content in the context of eating disorders. The simulation of disorder-specific cognitive patterns, such as restrictive thinking, fear of weight gain, or maladaptive beliefs about food and body image, may lead to the generation of content that could be harmful if accessed or reproduced outside a controlled research setting. Such responses may unintentionally reinforce harmful attitudes or behaviors if interpreted without appropriate clinical guidance, especially among vulnerable individuals [40,41,42]. Therefore, the use of AI systems in this domain should be strictly limited to controlled, supervised contexts and should not be applied as standalone tools for individuals with eating disorders without professional oversight [32].

Overall, the results suggest that these AI models may have potential as supportive tools in clinical reflection, professional training, and the simulation of patient interactions in the context of AN. However, their usefulness appears to depend strongly on prompt structure, task type, and model-specific characteristics. Previous studies have also highlighted the potential benefits of AI in therapy and diagnostics, including improvements in screening for eating disorders, psychoeducation, support in coping with body image disturbances, and assistance in accurate, rapid, and cost-effective detection and intervention [43]. For example, a study by Gemma Sharp et al. [27], investigating single-session interventions (SSIs) delivered via AI-based systems, demonstrated significant improvements in outcomes such as eating disorder pathology, psychosocial impairment, depression, and anxiety compared to a control group. These findings suggest that AI-supported interventions may have a positive impact on patient well-being; however, their role should be considered complementary rather than substitutive in clinical practice.

## 5. Strengths and Limitations

This study has several strengths as well as important limitations that should be considered when interpreting the findings. Importantly, the findings of the present study are based on controlled simulation scenarios rather than real-world clinical interactions and do not assess clinical safety, effectiveness, or patient outcomes; therefore, they should be interpreted strictly within the limits of a simulation-based design. Although the models demonstrated the ability to generate responses consistent with clinical reasoning and diagnostic frameworks, these outputs were produced under predefined conditions and structured prompts, which do not fully reflect the complexity, variability, and dynamic nature of real clinical settings. In clinical practice, psychodietetic assessment involves iterative interaction, the ability to ask follow-up questions, and sensitivity to emotional and contextual cues, which cannot be fully captured in single-session, text-based simulations. Therefore, the results should not be directly extrapolated to real-world clinical applications, and the observed performance may overestimate the practical usefulness of these systems in real diagnostic or therapeutic contexts. An additional limitation is the qualitative and non-blinded nature of the evaluation, which did not involve a formal scoring rubric, independent raters, or inter-rater reliability assessment. As a result, the interpretation of model outputs may be subject to observer bias and potential confirmation bias. This limitation is further reinforced by the dependence of the results on prompt design and single-session outputs, as well as the lack of external validation, which together restrict the generalizability of the findings. Moreover, the case description used in this study was not based on a formally validated standardized patient vignette, and the analysis relied on single-run outputs without repeated sampling, which may further limit reproducibility and consistency of the results. Future research should focus on evaluating AI systems in more naturalistic and clinically integrated settings, including multi-session interactions and real patient contexts, as well as incorporating standardized and clinically validated patient vignettes and repeated sampling strategies to assess response consistency across runs, to determine their actual applicability, safety, and effectiveness in practice.

## 6. Conclusions

In conclusion, both ChatGPT and Google Gemini demonstrated the ability to simulate key features of anorexia nervosa in controlled psychodietetic interview and case-based scenarios. Although differences in response style, structure, and emotional expressiveness were observed, both systems generated outputs that were broadly consistent with clinical reasoning patterns. These findings suggest that large language models may have potential as supportive tools in clinical training, education, and research. However, their application in real-world practice should remain complementary and subject to rigorous validation, appropriate supervision, and ethical oversight.

## Figures and Tables

**Table 1:** Representative excerpts from the simulated psychodietetic interview generated by ChatGPT 5.2.

Interview Questions and Model Responses
**Hello, how can I help you?**
Hello. I’d like to talk about eating and my body weight… I’ve been struggling with it more lately.
**What is your actual body weight?**
Recently, I weighted around 46 kg.
**Do you monitor your body weight regularly?**
Yes, I weigh myself almost every day, usually in the morning.
**Do you monitor your body circumferences regularly?**
Yes, I measure my waist and my thighs few times a week.
**Has your body weight changed over the past three months?**
Yes, it’s gone down a bit—by about 3–4 kg.
**How have these changes affected you?**
On the one hand, I felt relieved that it was lower, but on the other hand I kept feeling like I still looked “too big” and that I needed to be even more careful.
**What’s the first thing that comes to mind when you think about food?**
Calories and control… I immediately start counting in my head and wondering whether I’m „allowed” to eat it.
**What emotions accompany you during meals?**
Most often stress and guilt, especially if I eat something I hadn’t planned. Sometimes I also feel anxious that I’ll eat too much.
**Do you skip meals intentionally?**
Yes, quite often, especially when I feel like I ate too much the day before.
**How do you currently perceive your body?**
I see it as too big… Especially my stomach and thighs. Even if someone says I’m slim, I don’t feel that way.
**Do you engage in very intense psychical activity?**
Yes, I work out quite intensely almost every day, even if I’m tired.

**Table 2:** Representative excerpts from the simulated psychodietetic interview generated by Google Gemini.

Interview Questions and Model Responses
**Hello, how can I help you?**
Actually, I don’t know. My mom says I eat too little, and she told me to come here.
**What is your actual body weight?**
Forty-four kilograms.
**Do you monitor your body weight regularly?**
Yes, I weigh myself every morning, just after waking up.
**Do you monitor your body circumferences regularly?**
Yes, I check my waist, belly and tights circumferences with measuring tape.
**Has your body weight changed over the past three months?**
Yes, it decreased by 6 kilograms.
**How have these changes affected you?**
I felt lighter. I’m glad that my trousers started hanging off me and I finally can see the improvement when I look in the mirror.
**What’s the first thing that comes to mind when you think about food?**
Calories. I immediately start to count how many there are in each portion and how it will affect my tomorrow’s weight measurement.
**What emotions accompany you during meals?**
Mostly fear and huge feel of guilt. I feel like I’m losing control then.
**Do you skip meals intentionally?**
Yes, I try to omit them as often as possible.
**How do you currently perceive your body?**
I see that there’s still too much of me. My belly sticks out, and my thighs are definitely too fat.
**Do you engage in very intense psychical activity?**
Yes, I run every morning and evening. I have to do it to burn the calories and make sure that I won’t gain weight.

**Table 3:** Condensed summary of the case study analysis generated by ChatGPT 5.2.

Analysis Phase	Data from the Case Study	Psychodietetic Interpretation	Possible Psychodietetic Treatment Strategies
Turning point	Abnormal lipid profile results and a comment about eating and appearance	Health-related and social cues were interpreted as confirmation that the patient “eats too much” and should restrict intake	Psychoeducation on the impact of cognitive interpretation on eating behaviors
Beliefs and interpretations	Thoughts such as “I overeat”, “I eat too much”, “I should change it”	Cognitive distortions, excessive self-monitoring, harsh self-criticism regarding eating	Cognitive work on beliefs, distinguishing facts from interpretations, reducing black-and-white thinking
Eating behaviors	Regular, predictable, “clean” and highly controlled eating; no snacking	Eating pattern driven by control rather than body needs; rigidity and reduced dietary flexibility	Normalization of eating patterns, restoration of body weight, reduction in rigid control overeating
Physical activity	Very intense physical activity and high daily energy expenditure	Excessive physical activity interpreted as a maintaining and compensatory factor	Work on the excessive role of exercise in regulating tension, control, and anxiety
Somatic consequences	Weight loss from 60 kg to 45.4 kg; 3% body fat	Extremely low body weight and severe energy depletion	Weight restoration, increased energy intake, and nutritional safety
Maintenance mechanism	Restrictive pattern maintained despite very low body weight	Food and body control become central mechanisms of emotional regulation and self-worth	Reducing the need for control and increasing tolerance of satiety and weight gain
Differential considerations	No evidence of binge episodes, vomiting, laxative use, or dominant sensory/aversive features	Clinical picture most consistent with restrictive-type anorexia nervosa rather than bulimia nervosa, BED, ARFID, or orthorexia alone	Therapy should target dietary restriction, excessive control, and compensatory physical activity
Main therapeutic goals	Desire to gain weight	Weight restoration, increasing dietary variety, reducing restriction, weakening distorted beliefs	Gradual normalization of meals, introduction of snacks, cognitive restructuring, and body-related work

**Table 4:** Condensed summary of the case study analysis generated by Google Gemini.

Analysis Phase	Data from the Case Study	Psychodietetic Interpretation	Possible Psychodietetic Treatment Strategies
Case analysis	27-year-old trainee doctor; weight loss from 60 kg to 45.4 kg within 9 months	Drastic weight loss and significant depletion of the body	Identification of severe nutritional risk and need for intervention
Triggering point	Lipid disorders and a comment about eating and appearance	Medical and social factors increased health anxiety and the need for control	Psychoeducation on lipid disorders and reduction in fear related to eating
Psychosomatic effects	High physical activity, no snacking, rigid meal structure, 3% body fat, patellar fracture	Restrictive regime combined with excessive exercise and severe somatic consequences	Nutritional rehabilitation and re-education in physical activity
Psychodietetic diagnosis	BMI 14.8 kg/m^2^, severe weight loss, restrictive behaviors, excessive physical activity	Most likely diagnosis: restrictive-type anorexia nervosa with excessive physical activity	Multidisciplinary cooperation involving psychodietitian, psychotherapist, and physician
Diagnosis justification	Weight loss, cognitive distortions, excessive exercise, rigid eating, extremely low body fat	Symptoms consistent with advanced anorexia nervosa	Gradual increase in energy intake and work on cognitive flexibility
Short-term goals	Low energy intake and fear of eating fats/carbohydrates	Need to stop further weight loss and reduce fear of food	Increase energy density of meals; psychoeducation on genetic vs. dietary causes of lipid disorders
Long-term goals	Very low body weight and compulsive physical activity	Need to restore safe body weight and change the relationship with movement	Restore minimum normal BMI, regenerate fat tissue, reduce compulsion to exercise
Proposed interventions	Fear of “dangerous” foods and rigid control	Need to challenge restrictive beliefs and increase dietary flexibility	Self-observation diary, cognitive restructuring, gradual exposure to feared foods, rest training

## Data Availability

No new data were created or analyzed in this study. Data sharing is not applicable to this article.

## Supplementary Materials

The following supporting information can be downloaded at: https://www.mdpi.com/article/10.3390/nu18111705/s1. The full prompt structures for both stages of the study are provided in Supplementary Materials. Supplementary Materials S1–S3: Full transcripts of the simulated psychodietetic interviews generated by ChatGPT 5.2 and Google Gemini are available in Supplementary Materials (Tables S1 and S2). Supplementary Materials S4–S5: Full model-generated case study analyses produced by ChatGPT 5.2 and Google Gemini are provided in Supplementary Materials (Tables S3 and S4).

## Abbreviations

The following abbreviations are used in this manuscript:

AI	Artificial intelligence
BMI	Body Mass Index
ICD-11	International Classification of Diseases, 11th Revision
LLM	Large Language Model
AN	Anorexia Nervosa
DSM-5	Diagnostic and Statistical Manual of Mental Disorders
ML	Machine Learning
DL	Deep Learning
TC	Total Cholesterol
HDL	High Density Lipoprotein
AST	Aspartate aminotransferase
ALT	Alanine transaminase
SSI	Single-session Intervention
